# Glycemic control, including Time in Tight Range (TITR) evaluation, in children with type 1 diabetes treated with the MiniMed 780G system: a 3-year prospective, observational, two-center study

**DOI:** 10.3389/fendo.2026.1750164

**Published:** 2026-02-13

**Authors:** Mateusz Tarasiewicz, Wiktoria Bartnikowska, Agata Chobot, Anna Bielawska, Agnieszka Zając, Joanna Polańska, Przemysława Jarosz-Chobot, Sebastian Seget

**Affiliations:** 1 Department of Children’s Diabetology and Lifestyle Medicine, Medical University of Silesia, Katowice, Poland; 2 Department of Pediatrics, Institute of Medical Sciences, University of Opole, Opole, Poland; 3 Department of Data Science and Engineering, Silesian University of Technology, Gliwice, Poland

**Keywords:** automated insulin delivery, children, diabetes, diabetes mellitus, time in tight range, type 1 diabetes

## Abstract

**Background:**

The MiniMed 780G was the first commercially available automated insulin delivery (AID) system in Poland. The aim of this study was to analyze glycemic management in children with type 1 diabetes (T1D) after three years of using the system.

**Materials and methods:**

The data included anthropometric measurements, information from insulin pumps, and continuous glucose monitoring (CGM) from fifty children with T1D (mean age: 9.9 ± 2.4 years; T1D duration at baseline: 3.9 ± 2.56 years) using the MiniMed 780G. The data were collected at AID initiation, 12 and 36 months after starting its use.

**Results:**

Coefficient of variation (CV) and body mass index (BMI) z-scores at baseline and after 3 years were comparable (p > 0.05). TITR firstly increased after the AID system implementation with the peak value after 6 months (60.82% ± 9.34), then the values decreased to 56.50%± 10.78% after 3 years (p<0.001). Time in range (70–180 mg/dL) decreased after 3 years of observation compared to peak values at 6 and 12 months (77.74% ± 7.65% versus 81.16% ± 7.83% and 80.40% ± 8.25%, respectively, p<0.005. The percentage of auto-correction in total daily insulin dose increased significantly (11.24% ± 6.08% 2 weeks of AID system usage vs. 16.34% ± 6.69% after 3 years; p < 0.001).

**Conclusions:**

Despite a slight deterioration after 3 years, optimal glycemic management was maintained throughout the observation period. GCM metrics remained within recommended values in early adolescence, most likely due to auto-correction boluses.

## Introduction

T1D, as the most common type of glucose metabolism disorder in the pediatric population, remains an enormous challenge for young person, their caregivers and healthcare professionals. The incidence of new-onset T1D in children and adolescents is still rising (1).

Through the decades, the therapy standards have changed, and modern insulin delivery systems are still one of the most promising achievements of contemporary medical technology. In 2025, there is strong evidence that the AID technology changed the everyday lives of children and adolescents with T1D, in terms of maintenance of stable glycemia, as well as in the field of quality of life (2–6). The use of AID technology also created a new reality of well-being for caregivers (7, 8). The difficulties specific to the pediatric population change with the age of the person. Early childhood as well as adolescence seem to be one of the most challenging periods. In the latter, T1D treatment needs to take into account the hormonal changes during puberty, the emotional fluctuations and potential risk behaviours.

We present a 3-year prospective, observational, two-center study, a follow-up of two research studies based on the same pediatric population treated with the MiniMed 780G system, published in 2022 and 2024 (2, 3). The current analysis focused on CGM data and anthropometric data of children with T1D using this AID system for the last 3 years and is the first such extended observation of the use of the MiniMed 780G system in the pediatric age group.

## Materials and methods

The Minimed 780G was the first approved AID system in Poland (9). We followed prospectively 50 children with T1D using the MiniMed 780G system, who remained under the care of two pediatric diabetes centers in southern Poland (Department of Children’s Diabetology and Lifestyle Medicine, University Clinical Hospital of the Medical University of Silesia in Katowice and Department of Pediatrics, University Clinical Hospital of the University of Opole). Treatment in both centers follows the same standards of care for children with T1D as per the guidelines of the International Society for Pediatric and Adolescent Diabetes [both are Centers of Reference of SWEET (Better control in Pediatric and Adolescent diabeteS: Working to crEate CEnTers of Reference)] and annually updated guidelines by Diabetes Poland (10). All of the children participating in this study used a sensor-augmented pump with predictive low glucose suspend (SAP-PLGS) before starting the AID system. The inclusion criteria for the observation group were age ≦18 years, more than 70% of the CGM wear and usage time, and more than 70% of time spent in the automatic mode after starting AID (for adequate interpretation of the AID system data). Most of the children used the optimal settings of the MiniMed 780G - active insulin time (AIT) 2 hours and target glucose 100 mg/dl. Age, sex, duration of T1D, body weight and height were for each individual were collected prospectively at several time points: right after AID implementation, then 6, 12, 24 and 36 months afterwards. For each time point, we calculated the BMI z-score using the participant’s weight and height, following the World Health Organization (WHO) reference values (11). We also collected the HbA1c values at the baseline and after 36 months of follow-up. Data from the AID system was automatically sent to the CareLink server and retrieved using CareLink Professional software (Medtronic MiniMed, USA). CGM data were analysed using GlyCulator 3.0 software (thanks to Medical University of Łódź, Poland) (12). Two-week AID data were collected prospectively at the same time points as mentioned above, including average sensor glucose (AvgSG), glucose management indicator (GMI), coefficient of variation (CV), time in range (TIR) 70–180 mg/dl, time in tight range (TITR) 70–140 mg/dl, time above range (TAR) > 250 mg/dl, TAR 180–250 mg/dl, time below range (TBR) 54–70 mg/dl, TBR < 54 mg/dl, total daily insulin (TDI), percentage of insulin boluses and basal insulin, percentage of autocorrection usage, percentage of time using CGM, and percentage of time using SmartGuard. For each variable, the standard descriptive statistics (mean value, standard deviation, and their 95% confidence intervals, range, minimum and maximum values, quartiles, skewness and kurtosis) were estimated. The Lilliefors test was applied to check the normality of distribution within each endpoint. Friedmann’s ANOVA test for repeated measures with Nemenyi *post hoc* tests were applied to detect changes in time responses. Kendall’s concordance coefficient W was calculated to quantify agreement in time responses across patients and estimate the effect size (13). The Jonckheere-Terpstra test for trend was used to recognise significant linear patterns in time responses. The Benjamini-Hochberg estimates of the false discovery rate (FDR) were calculated to accompany the p-values.

## Results

**Figure 1:**
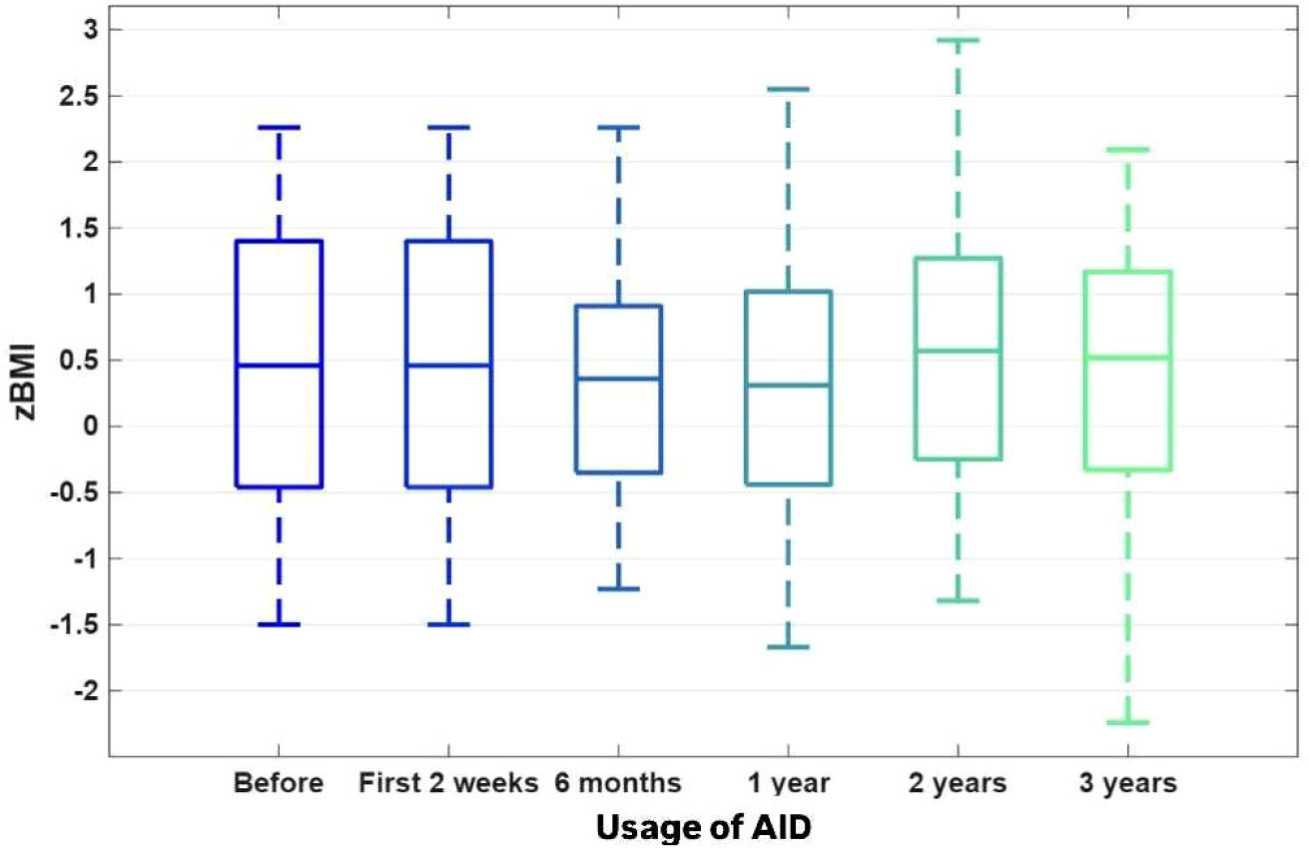
The BMI Z-scores.

The baseline mean age of the 50 children (24 [48%] boys) was 9.9 ± 2.4 years, and the mean duration of T1D was 3.9 ± 2.56 years. BMI z-scores showed heterogeneous fluctuations over time (W = 0.05), with an upward trend observed during the initial 24-month period (Friedman ANOVA p = 0.043789). The significant increase observed during the first 24 months was subsequently counterbalanced, resulting in values at 36 months that were comparable to baseline (p = 0.818202) (**Figure 1**).

**Figure 2:**
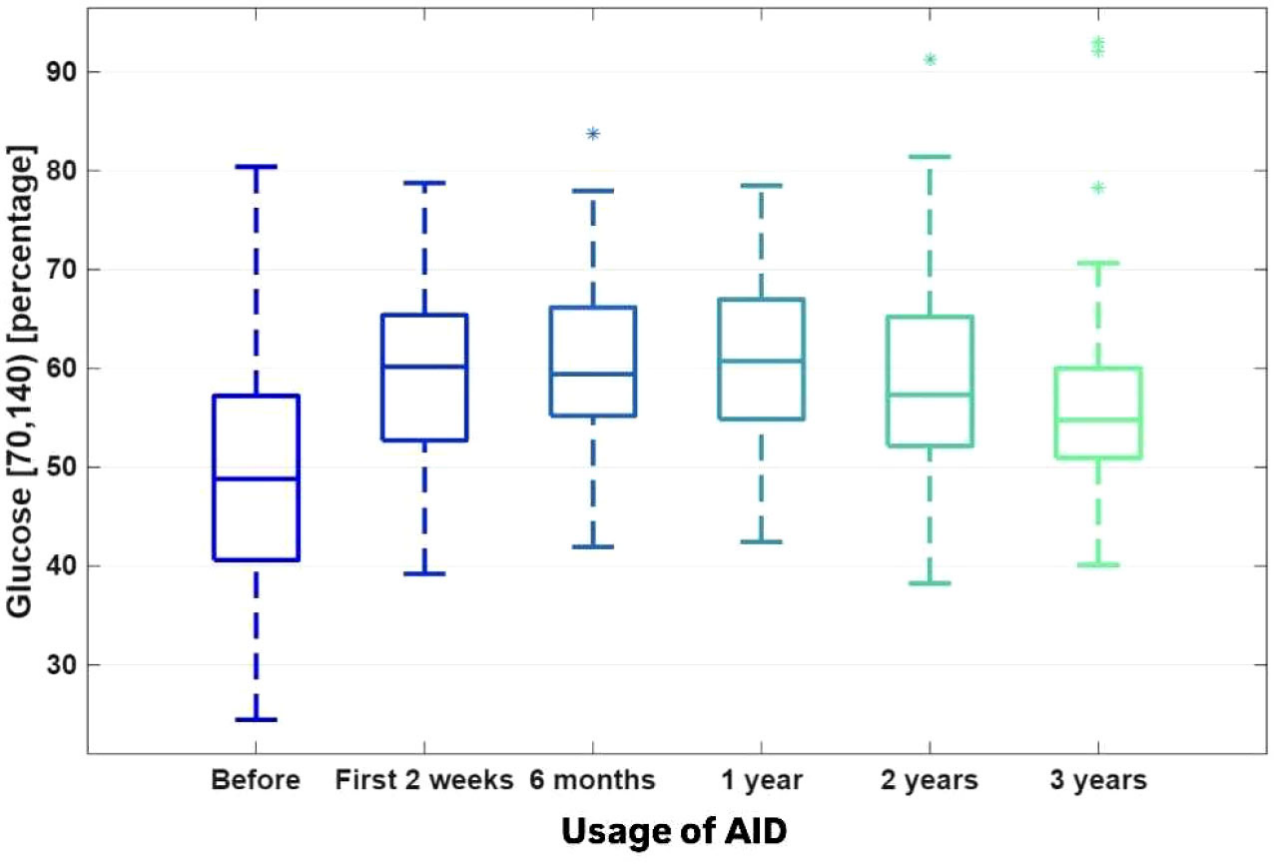
The TITR (time in tight range) 70–140 mg/dl [%]. **Figures 1**–**4**. The boxplots for the CGM data, BMI Z-score and TDI values before the implementation of the system, 2 weeks after the initiation of the AID system (Minimed 780G), and after 6, 12, 24 and 36 months of follow-up. The data after 3 years were compared to the previous values after 1 and 2 years of observation, which were presented by Seget S. et al. (3)(4).

TITR increased after AID system implementation, reaching a peak after 6 months (60.82% ± 9.34%; Friedman ANOVA p = 0.000001, FDR < 0.000001), followed by a decrease to 56.50% ± 10.78% after 3 years (p = 0.079950) (**Figure 2**).

**Figure 3:**
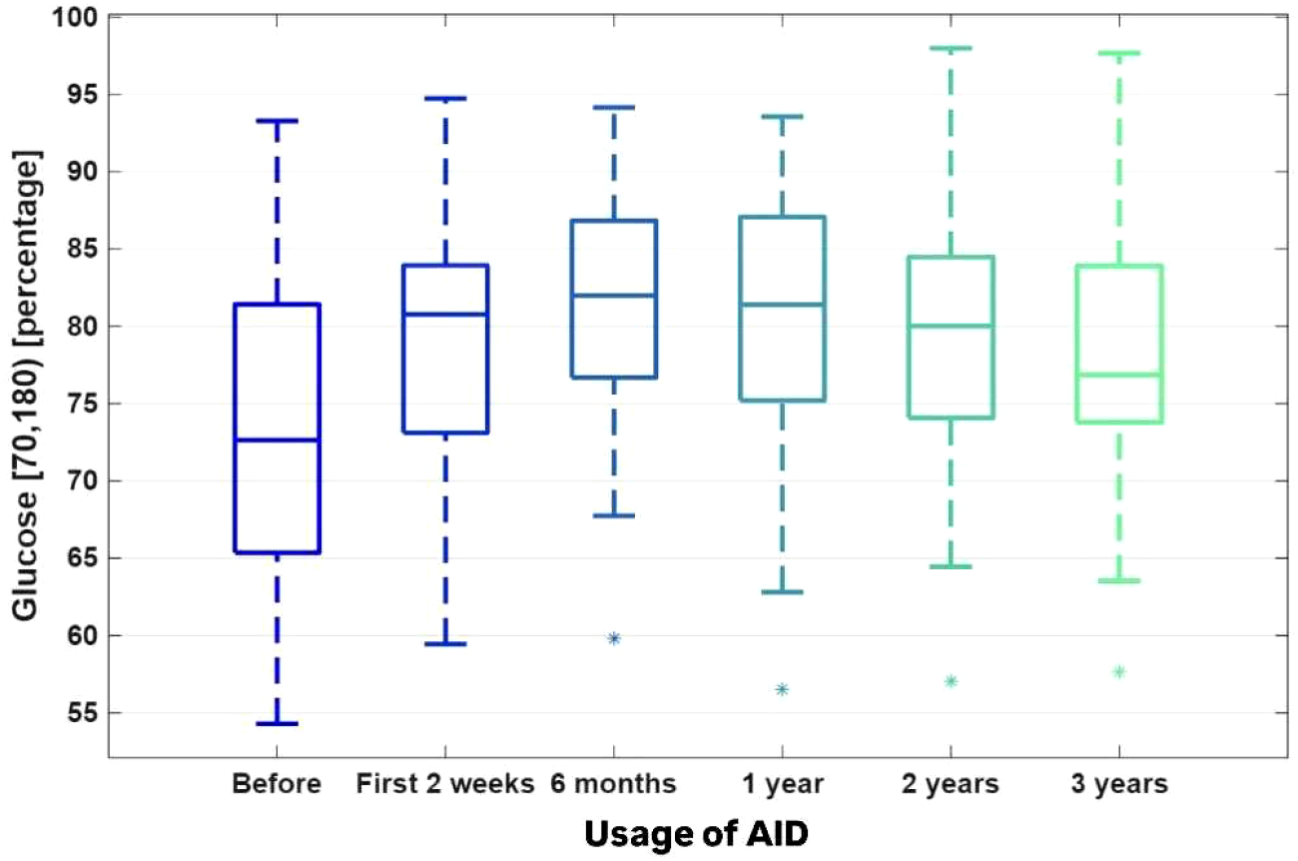
The TIR (time in range) 70–180 mg/dl [%].

TIR changed significantly over the 3-year observation period (Friedman ANOVA p = 0.000001, W = 0.14, FDR = 0.000002). The value at 36 months (77.74% ± 7.65%) was significantly lower than the peak value observed at 6 months (81.16% ± 7.83%; p = 0.030933) (**Figure 3**). Both TIR and TITR demonstrated statistically significant improvement over the first 2 years compared with baseline values (p < 0.05).

In addition, a statistically significant downward trend was observed for both TBR categories (Friedman ANOVA p < 0.05, FDR < 0.05). Baseline TBR values of 1.11% ± 1.07% for glucose <54 mg/dL and 4.15% ± 2.70% for glucose 54–70 mg/dL decreased significantly to 0.49% ± 0.52% and 2.50% ± 1.69%, respectively.

An unfavorable increase in TAR was observed during the final observation period. Both TAR 180–250 mg/dL and TAR >250 mg/dL reached values at 36 months comparable to those observed before initiation of AID therapy (p > 0.05). The time spent in the 180–250 mg/dL range increased significantly after 3 years compared with values after 1 year (p < 0.05). Additionally, the time spent at glucose levels >250 mg/dL showed a significant increase after 3 years of AID use compared with the periods after the first 2 weeks and after 1 year (p < 0.05).

**Figure 4:**
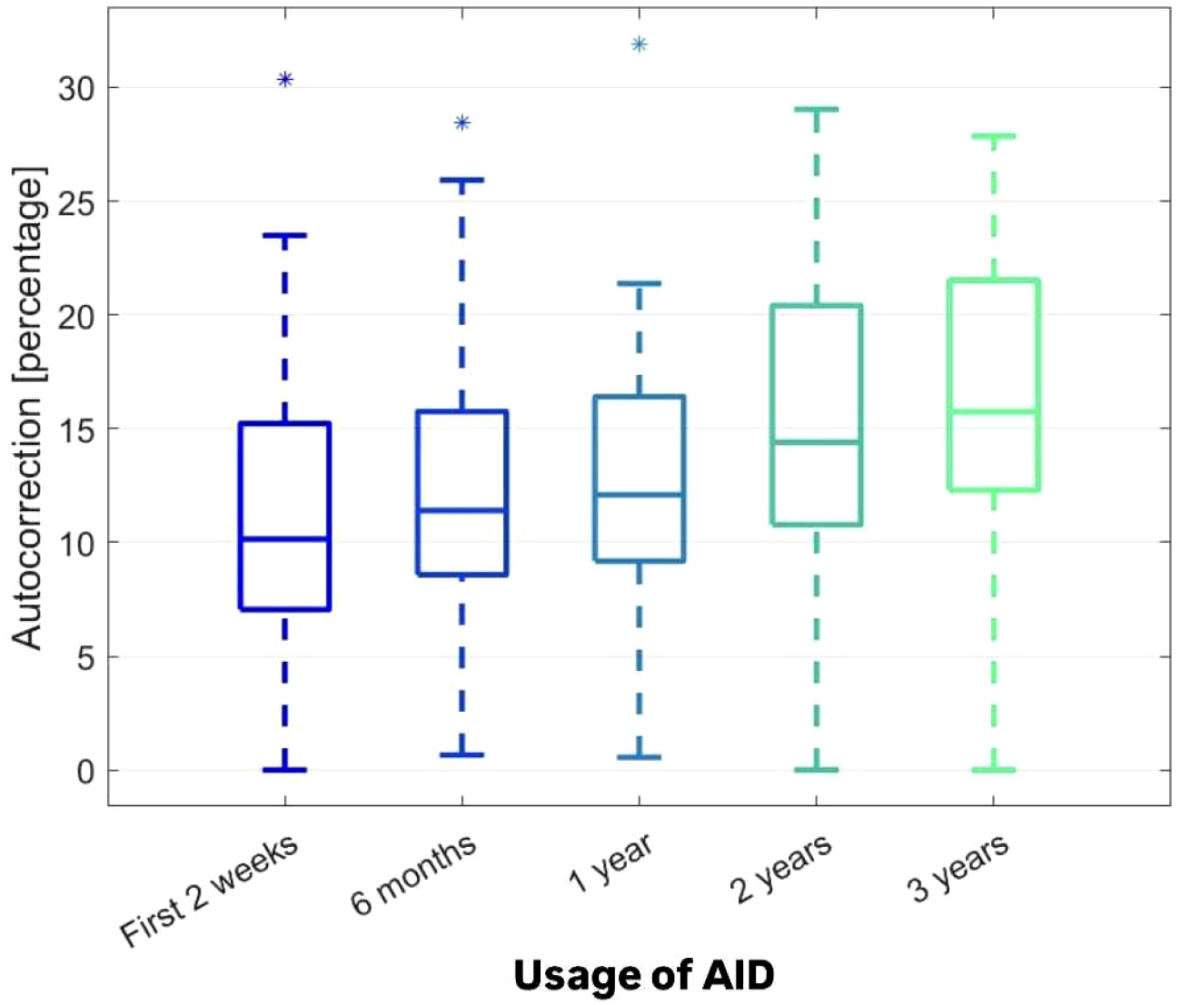
The autocorrection usage [%].

**Figure 5:**
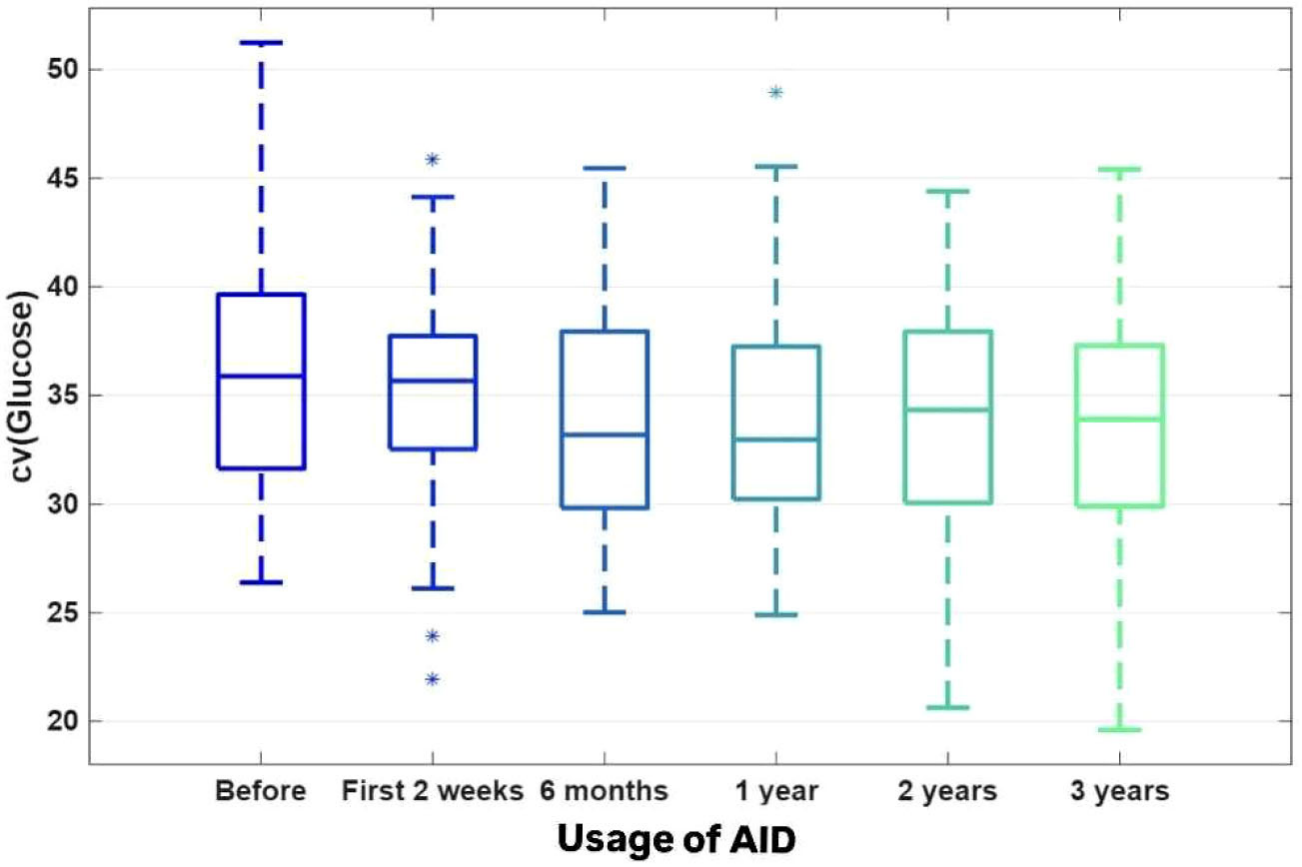
The coefficient of variation (CV) [%].

Concurrently, a consistent and significant increase was observed in the proportion of auto-correction within the total daily insulin (TDI) dose (Friedman ANOVA p < 0.000001, FDR < 0.000001) (**Figure 4**). Despite changes in most parameters over time, the CV remained unchanged (Friedman ANOVA p = 0.238656, FDR = 0.238655) (**Figure 5**).

A significant increase in sensor usage was observed from baseline throughout the 3 years of AID therapy (Friedman ANOVA p < 0.000001, FDR < 0.000001).

**Figure 6:**
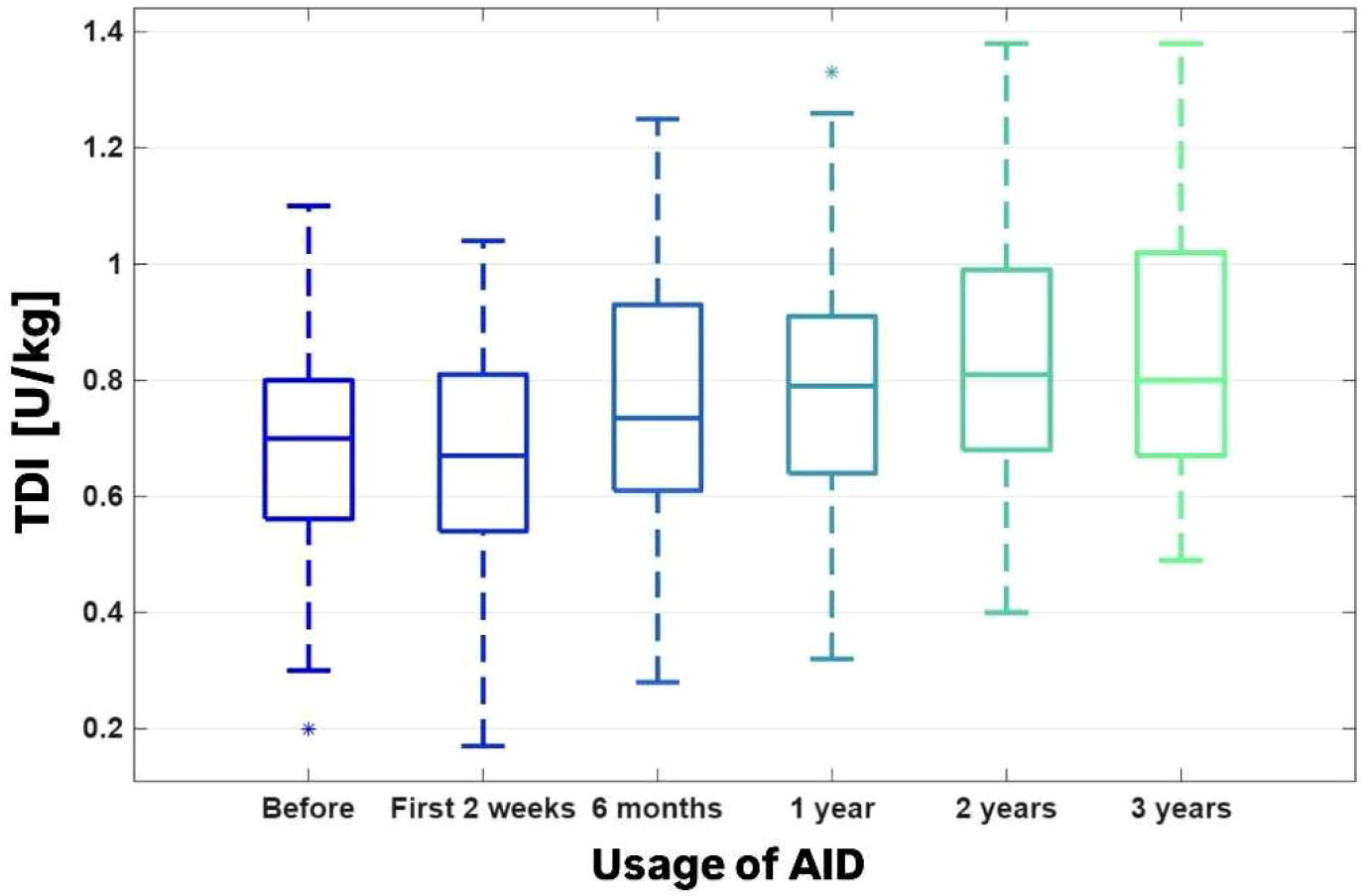
The TDI [U/kg].

TDI (U/kg) after 2 weeks of AID usage was comparable to the baseline values (0.69 ± 0.19 vs. 0.67 ± 0.20, p > 0.05), then it starts to increase, and according to the baseline it was statistically significant change in TDI value after 3 years of observation (0.69 ± 0.19 vs. 0.84 ± 0.22, p<0.01). (**Figure 6**).

The summarized data are presented in **Table 1**.

**Table 1:** Daily insulin doses, HbA1c, the AID system and CGM data of children and adolescents with type 1 diabetes using the advanced hybrid closed-loop system before the implementation of the Minimed 780G system, 2 weeks after the initiation of the system and after 6, 12, 24 and 36 months of follow-up.

The assessed parameter	The 2 weeks before AID implementation	The first 2 weeks of AID start	After 6 months	After 12 months	After 24 months	After 36 months	Friedman ANOVA p -value (P_A_) Jonckheere-Terpstra test p-value (P_T_) Kendall concordance coefficient (W) Benjamini_Hocheber FDR
Avg SG [mg/dl]	145.03 ± 15.93	131.36 ± 11.04	132.46 ± 11.73	132.45 ± 13.42	136.09 ± 13.62	139.70 ± 13.69	P_A_<0.000001 P_T_=0.030114 W=0.19 FDR<0.000001
GMI [%]	6.68 ± 0.46	6.45 ± 0.26	6.48 ± 0.28	6.48 ± 0.32	6.57 ± 0.33	6.65 ± 0.33	P_A_=0.00007 P_T_=0.260659 W=0.13 FDR=0.000012
HbA1C [%]	-	6.69 ± 0.65	-	-	-	6.57 ± 0.61	P_A_=0.101050 W=0.05
CV [%]	35.91 ± 5.72	34.99 ± 5.17	33.75 ± 5.02	34.06 ± 5.38	33.93 ± 9.86	33.77 ± 5.56	P_A_=0.238656 P_T_=0.022519 W=0.03 FDR=0.238655
TDI [u/kg]	0.69 ± 0.19	0.67 ± 0.20	0.77 ± 0.22	0.79 ± 0.20	0.84 ± 0.22	0.84 ± 0.22	P_A_<0.000001 P_T_=0.000054 W=0.33 FDR<0.000001
Carbohydrates [g]	193.24 ± 78.19	184.74 ± 78.42	206.44 ± 83.04	203.10 ± 79.80	215.20 ± 97.02	220.64 ± 113.10	P_A_=0.000646 P_T_=0.033939 W=0.09 FDR=0.000944
Basal insulin [%]	37.63 ± 10.83	34.88 ± 6.91	33.16 ± 5.09	35.08 ± 6.36	34.82 ± 6.21	34.65 ± 6.91	P_A_<0.000001 P_T_=0.000023 W=0.45 FDR=0.049195
Bolus insulin [%]	61.82 ± 10.97	65.06 ± 6.90	66.84 ± 5.09	64.92 ± 6.36	65.17 ± 6.21	65.12 ± 6.23	P_A_<0.000001 P_T_<0.000001 W=0.68 FDR=0.039648
Autocorrection [%]	–	11.24 ± 6.08	11.94 ± 5.63	12.40 ± 5.79	14.61 ± 7.00	16.34 ± 6.69	P_A_<0.000001 P_T_=0.000001 W=0.24
Usage of the sensor [%]	90.52 ± 8.25	95.10 ± 3.94	94.04 ± 4.69	93.80 ± 4.53	95.66 ± 3.66	96.02 ± 3.60	P_A_<0.000001 P_T_=0.000909 W=0.18 FDR<0.000001
Auto mode [%]	–	94.26 ± 6.28	96.62 ± 5.70	97.56 ± 2.79	97.58 ± 3.21	97.16 ± 4.12	P_A_=0.009132 P_T_=0.003643 W=0.07
TAR 250 mg/dl [%]	4.45 ± 3.51	2.33 ± 2.52	2.34 ± 2.31	2.68 ± 3.48	3.10 v 3.11	3.43 ± 3.15	P_A_=0.000012 P_T_=0.027718 W=0.12 FDR=0.000020
TAR 180–250 mg/dl [%]	18.05 ± 7.07	13.13 ± 5.74	12.83 ± 5.88	12.59 ± 5.78	14.21 ± 6.16	15.70 ± 5.51	P_A_<0.0000001 P_T_=0.008552 W=0.19 FDR<0.000001
TIR 70–180 mg/dl [%]	73.12 ± 9.77	79.28 ± 8.12	81.16 ± 7.83	80.40 ± 8.25	79.33 ± 8.19	77.74 ± 7.65	P_A_=0.000001 P_T_=0.000991 W=0.14 FDR=0.000002
TITR 70–140 mg/dl [%]	50.40 ± 11.38	59.45 ± 8.97	60.82 ± 9.34	60.72 ± 8.86	58.85 ± 10.36	56.50 ± 10.78	P_A_<0.000001 P_T_=0.00200 W=0.23 FDR<0.000001
TBR 54–70 mg/dl [%]	3.26 ± 2.42	4.15 ± 2.70	2.95 ± 1.76	3.40 ± 2.34	2.79 ± 2.09	2.50 ± 1.69	P_A_=0.013111 P_T_=0.077443 W=0.06 FDR=0.017794
TBR <54 mg/dl [%]	1.12 ± 1.63	1.11 ± 1.07	0.73 ± 0.77	0.93 ± 0.92	0.58 ± 0.70	0.49 ± 0.52	P_A_=0.026647 P_T_=0.082377 W=0.05 FDR=0.033753

ID, automated insulin delivery using Minimed 780G System; AvgSG, average sensor glucose; GMI, glucose management indicator; CV, coefficient of variation; TDI, total daily insulin; TAR, time above range; TIR, time in range; TITR, time in tight range; TBR, time below range. The measurements are represented as the mean value ± standard deviation.

## Discussion

The current study supports and adds interesting data to the former results of the previous observations of this group of children - after 12 and 24 months of using the AHCL system (2, 3).

Although TIR and TITR decreased slightly after the third year of observation, TIR and TITR remained in recommended ranges. The change in TIR was not associated with an increase in hypoglycemia, as both TBR 54–70 mg/dl and < 54 mg/dl values remained low. Both TARs increased in comparison to the previous time points after the initiation of the system, and it was correlated with a stable CV value and increase in AID usage time.

The TITR (70–140 mg/dl) is an emerging CGM parameter and was identified as an independent risk factor for the occurrence of acute and chronic complications of T1D (14–17). The association between TIR and TITR was shown to be nonlinear. TITR seems to better reflect the changes in CV and AvgSG in case of lower HbA1c values (18, 19).

Similar outcomes were presented in the study of 100,000 individuals treated with Minimed 780G in automatic mode. The authors observed a slight decrease in TIR over 12 months of the AID use (75.5% in the first month, vs. 73.3% in the next 12 months) (20). Interestingly, this decrease in our studied group occurred much later, in the third and not in the first year of AID use.

These findings could be attributed to several factors. People may become accustomed to the pump’s increased support in diabetes management and the “novelty effect” wearing off over time. Another issue could be the decrease in adherence to the orders of caregivers, which is commonly observed in the teenage group. The mentioned insights can be upheld by another crucial observation in the study. The percentage of auto-correction dose in TDI dose increased significantly with time of our observation. This shows that the children and their caregivers relied with time more on the AID system, but on the other hand, the users made less aware therapeutic decisions in their day-to-day life. Other data support the thesis that the use of autocorrection in the MiniMed 780G system allows a less restrictive adherence to the therapy (21).

Children setting out puberty can be less scrupulous in daily actions like carbohydrate counting or even entering carbs into the system. Carbohydrate counting is perceived as one of the most burdensome tasks in T1D and is frequently done inconsistently and with poor accuracy (22). It was shown that meal announcement by using a preset of three personalised fixed-carbohydrate amounts is a valuable alternative to the conventional carbohydrate counting and allows achieving international glycemic targets by most adolescents. Additionally, increasing age of the person with T1D was associated with more insulin delivered by autocorrection boluses due to fewer reported meals, more underestimated reported carbohydrates, and more possible changes in the amount of carbohydrates consumed (23). In the described group of age (mean age of studied participants was 9.9 ± 2.4 years) every simplification in insulin therapy which can lead to remaining optimal glycemic management seems to be helpful.

The dietary habits in children entering puberty change and the parental supervision decreases. Teenagers are prone to stick to an unbalanced diet based on high calories consumption from solid fats, added sugars and alcoholic beverages, also a significant decrease in the amount of consumed fruit and vegetables. Skipping meals is also a common behaviour. Unhealthy eating habits are associated with risky behaviors in adolescence, which can be an additional factor leading to deterioration of the glycemic outcomes (24). Studies highlight the need for parental involvement for improving glycemic outcomes, adherence to the recommended regimen, and the practice of self-management in adolescents with T1D (25).

Most of our study group entered adolescence during the 3rd year of observation. The decrease in TIR after 36 months compared to the values from the first year could result also from the physiological adversities related to puberty, such as insulin resistance (26–28).

To the best of our knowledge, this study is the first such long-term observation of children with T1D using the Minimed 780G system. The glycemic outcomes at baseline were satisfactory and quite homogenous within this group. The lack of data about the pubertal status of the investigated group of children is one of the limitations of the study. Other limitations include the lack of assessment of potential changes in dietary habits, the degree of independence in making therapeutic decisions, and the level of caregivers’ involvement in diabetes management throughout the observation time. Another limitation is a lack of patient-reported outcomes.

## Conclusions

The children and adolescents with T1D followed for 3 years while using the MiniMed 780G system, despite entering puberty, maintained recommended glycemic outcomes. Sustaining high TIR and TITR, with CV values in the recommended target, was accompanied by an increase of the percentage of auto-correction. It shows that throughout the observation period the users relied increasingly on the algorithm.

## Data Availability

The original contributions presented in the study are included in the article/supplementary material. Further inquiries can be directed to the corresponding author.
